# A Case of Eclampsia

**Published:** 1898-02

**Authors:** J. W. Price

**Affiliations:** Richland, Texas


For the Texas Medical Journal.

### A CASE OF ECLAMPSIA.

BY J. W. PRICE, M. D., RICHLAND, TEXAS.

I was called to see Mrs. B., primipara, aged 18 years, Dec. 9, 11:30 p. m. Found her with os uteri just beginning to dilate; said she had been in labor since 5 o'clock. After examining and finding every thing all right, I retired, telling them if needed to call me. Did not call me till breakfast. I examined her at 8 o'clock, Dec. 10. Os was not dilated very much. Some œdema of feet and legs; frequent micturition; highly colored urine; some albumin. Let her inhale chloroform between pains; pains were not frequent; one every eight to fifteen minutes.

Gave her quin. sulph. gr. xii. On making examination at 3 p. m., Dec. 10, found os dilated to the size of a silver dollar. At 4 p. m. she had her first convulsion; sent for Dr. B. in consultation. I wanted to empty the uterus as soon as practicable; he did not agree with me, but thought she would make it O. K. with the inhaling chloroform to the extent of surgical narcosis, to control convulsions and favor dilatation of the parturient canal.

The doctor left me with the "sack to hold," saying: "If the child is not born in a few hours, let me know, and we will deliver her." Dr. B. had not gone quite an hour when she had another convulsion. She had been given gr. xx of quin. sulph. I then gave her chloral hydrate and bromide of soda aa gr. 15, to be repeated every half hour until three doses were taken; then every three hours until she got full effect of the drug. At 10:30 o'clock convulsions were under control. She had been tormented with seven convulsions.

Sent for Dr. B. again; arrived just in time to see her have the eighth convulsion; os being sufficiently dilated, we delivered with forceps. Sometimes, when she had a convulsion, I would give her nitro-glycerine, gr. $\frac{1}{100}$, hypodermically, which seemed to cut the attack short. The child was born at 12 o'clock; uterus contracted and retracted nicely. Ordered her to take of bromidia a teaspoonful as often as needed to keep her quiet; had her to take quin. sulph. gr. xiij per day; moved bowels with castor oil and turpentine, of the former a tablespoonful, of the latter 15 drops; turpentine strips to abdomen.

She was also put on ℞ tr. nux vom. ʒss; seng ℥iv. M. Sig: Teaspoonful every six hours. Saw her four times; dismissed case Dec. 13; never has had any elevation of temperature.

Feeding for the first three days was mostly buttermilk and soups. Such are the results of my first case of eclampsia.

Success to the JOURNAL.

